# Low dose threshold for measuring cardiac functional metrics using four‐dimensional CT with deep learning

**DOI:** 10.1002/acm2.14593

**Published:** 2024-12-03

**Authors:** Pengwei Wu, Kyle Kim, Lauren Severance, Elliot McVeigh, Jed Douglas Pack

**Affiliations:** ^1^ GE Healthcare Technology & Innovation Center Niskayuna New York USA; ^2^ Department of Bioengineering, Medicine Radiology at University of California San Diego La Jolla California USA

**Keywords:** Cardiac CT, deep Learning, functional Analysis, low‐dose CT

## Abstract

**Background:**

Four‐dimensional CT is increasingly used for functional cardiac imaging, including prognosis for conditions such as heart failure and post myocardial infarction. However, radiation dose from an acquisition spanning the full cardiac cycle remains a concern. This work investigates the possibility of dose reduction in 4DCT using deep learning (DL)‐based segmentation techniques as an objective observer.

**Methods:**

A 3D residual U‐Net was developed for segmentation of left ventricle (LV) myocardium and blood pool. Two networks were trained: Standard DL (trained with only standard‐dose [SD] data) and Noise‐Robust DL (additionally trained with low‐dose data). The primary goal of the proposed DL methods is to serve as an unbiased and consistent observer for functional analysis performance. Functional cardiac metrics including ejection fraction (EF), global longitudinal strain (GLS), circumferential strain (CS), and wall thickness (WT), were measured for an external test set of 250 Cardiac CT volumes reconstructed at five different dose levels.

**Results:**

Functional metrics obtained from DL segmentations of standard dose images matched well with those from expert manual analysis. Utilizing Standard‐DL, absolute difference between DL‐derived metrics obtained with standard dose data and 100 mA (corresponding to ∼76 ± 13% dose reduction) data was less than 0.8 ± 1.0% for EF, GLS, and CS, and 5.6 ± 6.7% for Average WT. Performance variation of Noise‐Robust DL remained acceptable at even 50 mA.

**Conclusion:**

We demonstrate that on average radiation dose can be reduced by a factor of 5 while introducing minimal changes to global functional metrics (especially EF, GLS, and CS). The robustness to reduced image quality can be further boosted by using emulated low‐dose data in the DL training set.

## 1 INTRODUCTION

Coronary computed tomography angiography (CCTA) is a good prognostic test for patients with stable ischemia and is becoming more widely used.^1^, ^2^ While obtaining high‐resolution, high‐quality data for coronary vessel analysis, the same heartbeat can be used to obtain additional dose modulated views across the entire cardiac cycle for retrospective reconstruction and cardiac function analysis. Global longitudinal strain (GLS), circumferential strain (CS), radial strain, and ventricular dyssynchrony can all be measured in all chambers of the heart from these retrospective images.^3^, ^4^ Strain metrics of left ventricle (LV) function, such as GLS, have been shown repeatably to be superior to ejection fraction (EF) for prognosis of outcomes in ischemia and heart failure.^5^ Recently, dyssynchrony metrics of myocardial strain have been shown to improve prediction of response to cardiac resynchronization therapy.^6^ Myocardial perfusion can also be estimated if additional heartbeats are used to measure the dynamics of contrast enhancement in the myocardium.^7^, ^8^

However, this valuable information about cardiac function and perfusion requires additional dose. Initial studies using simple segmentation techniques have shown that low x‐ray flux is required to measure cardiac function^9^, ^10^ however, the question remains: Can radiation dose be significantly reduced while still allowing functional parameter estimates that are accurate? The work described in this paper answers this question using state‐of‐the‐art deep learning (DL) segmentation as an objective observer; we analyze the same clinical images at progressively lower doses through accurate noise injection to observe the threshold dose reduction at which the DL analysis of function parameters becomes unacceptable.

Recent advances in DL have opened new avenues in the analysis of 4D cardiac images.^11^ Using DL, we avoid the variability in function analysis derived from human guided segmentation. Images at different dose levels were obtained using a validated noise injection method: that is, lower dose images were emulated by modifying the clinical standard dose raw data with accurate quantum and electronic noise injection.^12^ Images were analyzed at five dose levels: standard x‐ray flux (∼500 mA), 100, 50, 25, and 10 mA. Note that we performed the analysis at the extremely low dose emulation of 10 mA to ensure we observed a level where functional analysis failed. Our intention was not to demonstrate that function analysis can work even at the lowest dose level studied in this manuscript.

In this work, we report the following: (i) a well‐trained DL approach that returns segmentations and cardiac function estimates that agree with those obtained by expert human guided analysis; (ii) the minimum dose level that obtains accurate cardiac functional parameter estimates. In addition, we demonstrate a performance boost when including emulated low‐dose data in DL training (compared to DL training with only standard dose data). Note again that the main purpose of this work was not to propose a DL segmentation method that we expect to be universally optimal for low dose CT. Instead, the primary goal of the proposed DL methods is to serve as an unbiased and consistent observer for functional analysis performance. An ancillary finding in this work was the improvement in the network performance after inclusion of low dose images in training.

## 2 MATERIAL AND METHODS

### 2.1 CT data collection

This Health Insurance Portability and Accountability Act (HIPPA)‐compliant retrospective study was approved by the institutional review board (IRB) with a waiver of informed consent. A total of 170 electrocardiographically (ECG) gated cardiac CT studies between March 2008 and August 2020 were retrospectively collected. Sixteen patients were excluded due to severe metal artifacts (e.g., from cardiac leads). Patients for the external test data (25 patients, detailed in Table 1) were selected if they exhibited clearly abnormal LV function, or clearly exhibited normal LV function on Cine CT. Patients were randomly curated for these dataset w/o targeting particular body types and/or pathologies.

**TABLE 1:** Demographic and technical information for the standard‐dose (SD) set, the low‐dose (LD) set, and the External Test Set.

	Standard‐dose (SD) set	Low‐dose (LD) set	External Test Set
# Patients	114	15	25
# Volumes	335	150	250
Age (years)	64.6 ± 15.5	64.6 ± 11.9	59.5 ± 14.2
Male (%)	52.5	57.1	45.2
Scan date	2008/03–2020/08	2018/11–2019/03	2018/09–2020/08
Dose levels	SD only, ∼500 mA	SD, 100, 50, 25, 10 mA	SD, 100, 50, 25, 10 mA
3D label?	Y	Y	N
Manual label?	N	N	Y

*Note*: Standard‐dose set was further divided into SD training set, SD validation set and SD internal test set with an 80%, 10%, and 10% random split.

From the selected CT studies we created three different datasets which were independently curated: (i) a standard‐dose (SD) set that consists only SD data, (ii) a low‐dose, (LD) set that consists of data from five different dose levels: one standard dose image (SD, ∼500 mA) and four low dose emulated versions of the same image (at 100, 50, 25, 10 mA) per patient, and (iii) an External Test Set that also consists data of five different dose levels per patient (same dose levels as the LD set). There was no patient overlap between these datasets. This grouping strategy was designed to mimic a realistic continuous learning workflow of DL models, where the network is first trained with common (i.e., SD) datasets, then tuned with task‐specific (i.e., LD) datasets, and finally tested on the targeted External Test Set. The SD set was further divided into the SD training set, the SD validation set, and the SD internal testing set with an 80%, 10%, and 10% random split for network development and hyperparameter tuning. Standard dose data from the External Test Set is referred to as the SD External Test Set. Demographic and technical information for these datasets is shown in Table 1. No additional data augmentation was performed in this work.

To avoid repeated patient scans and the corresponding dose level increase, the low‐dose data (i.e., 100, 50, 25, 10 mA) in LD and the External Test Sets were analytically emulated from the standard dose scan of the same patient using a physics‐based noise injection pipeline, which accurately inserts Poisson‐distributed quantum noise and Gaussian‐distributed electronic noise in the projection domain.^12^ Validation of emulation accuracy can be found in ref.[12] The institutional standard dose levels for patients included in this study lead to an average tube current of 503 ± 178 mA with BMI dependent kVp (BMI<25: 80 kVp, 25<BMI<40: 100 kVp, BMI>40: 120 kVp). Most of the acquisitions (∼60%) were performed at 100 kVp.

All studies in the External Test Set were acquired with a single wide‐coverage 256‐row CT [GE HealthCare Revolution (Waukesha, WI)] allowing for an axial acquisition of the entire heart within a single heartbeat. Two functional phases, end‐diastole (ED) and end‐systole (ES), were reconstructed for each patient using the vendor cardiac image reconstruction method with the imaging kernel set to “standard”.

### 2.2 Study workflow

**FIGURE 1:**
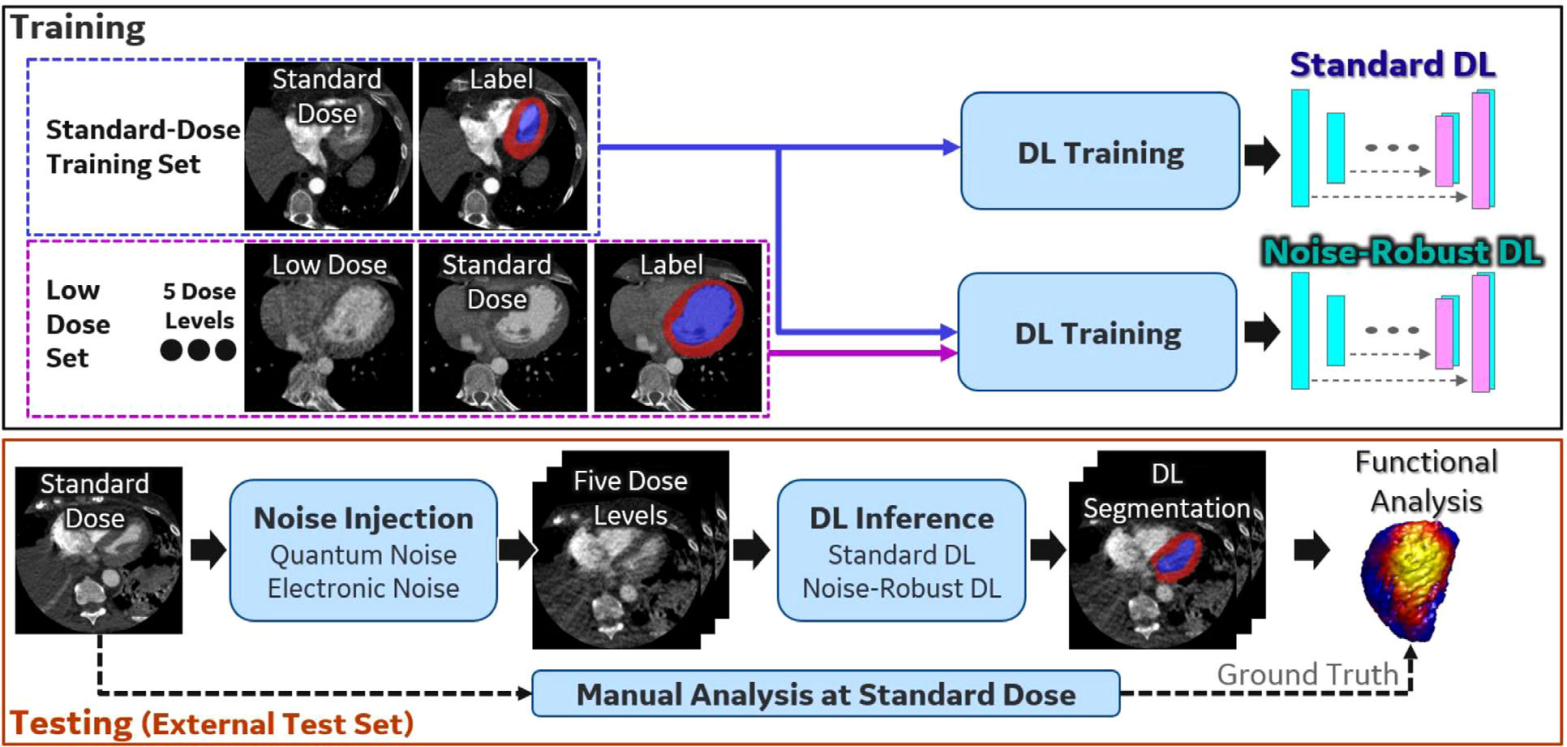
Workflow of the proposed DL‐based functional cardiac analysis pipeline. Two networks [(i) Standard DL, trained with only the standard dose training set; (ii) Noise‐Robust DL, trained with the standard dose training set and the low‐dose set] were developed and evaluated at five dose levels. Low‐dose data was created from the SD data with a physics‐based noise injection pipeline.^12^ Performance of DL was also evaluated against manual functional analysis of standard dose data from expert observers. DL, deep learning; SD, standard‐dose.

Figure 1 provides the schematic of the proposed training and testing pipelines for the DL‐based segmentation methods. Two DL networks, named “Standard DL” and “Noise‐Robust DL” were trained in this work using different sets of training data. Standard DL was trained with only the SD training set, while Noise‐Robust DL was trained with both the SD training set and the LD set. Both networks were applied to the external test data to measure performance against expert human observers. In addition, we measured the low dose threshold below which the segmentation and functional analysis performance was unacceptable; as mentioned above, an advantage of using a trained DL network for this work is to create a single efficient and objective observer to detect the low dose threshold. Getting human observer results at each dose levels can be time‐consuming and inconsistent, especially at low‐dose levels. Therefore, only DL observer (i.e., DL segmentation network followed by functional analysis) were used for low‐dose datasets. Quantitative cardiac functional metrics (EF, GLS, CS, and average wall thickening [WT]) were then derived from DL segmentations. We compared DL‐derived metrics with the same metrics obtained from expert human manual analysis of standard dose data and evaluated their robustness at different dose reduction levels.

### 2.3 DL implementation

A 3D U‐Net^13^ type of network, which features two residual blocks at each of the four encoder‐decoder levels, was used in this work. The deep supervision technique^14^ was additionally used to regularize segmentation accuracy and learned features at each resolution level. Generalized Dice loss function was used for the multi‐label segmentation (background, myocardium, and LV blood pool). Patch‐based (128 × 128 × 64) training was performed with the Adam optimizer (learning rate: 5 × 10^−4^, batch size: 32) for 200 epochs. Manual segmentation was performed by two observers (J.P., and K.K.) to provide 3D labels for the networks (referred to as “3D Label” in Table 1).^15^

### 2.4 Quantitative image analysis

In the original CT coordinate system, the voxelwise LV myocardium and blood pool segmentations were created by DL (Standard DL and Noise‐Robust DL). 3D Dice coefficient and EF were calculated within this coordinate system. Then, CT volumes and their corresponding DL segmentations were resampled into one 2 chamber long‐axis view (2‐CH), one parasternal long‐axis view (PLAX), and three short axis views (SAX). Three locations were used to sample the SAX views along the long axis: basal, middle (between base and apex), and apical. GLS was measured by averaging long axis strain / shortening in the 2‐CH and PLAX views.^16^ CS was measured in each of the three SAX views and reported separately. WT was measured at 16 radial directions equally spaced across 360°. Average WT was then calculated by averaging WT measurements across all radial directions and reported separately for each SAX view.

In this work, manual analysis of GLS, CS, and average WT was done by two expert observers (K.K., and E.M.) in Horos (referred to as “Manual Label” in Table 1), while automatic analysis of these metrics was done in MATLAB using resampled DL segmentation slices. Additionally, the observers also performed manual 2D segmentation for all three SAX views in Horos, making it possible to evaluate the same SAX views sampled from the 3D DL segmentations and compare the performance (i.e., 2D Dice coefficient). Observers J.P., K.K., and E.M., have 18, 3, and 34 years of experience reading cardiac CT images respectively.

## 3 RESULTS

Characteristics of the three datasets used in this work are shown in Table 1.

### 3.1 DL validation: Accuracy against manual labels

The validation of the DL networks versus manual analysis was evaluated with: (i) 3D Dice coefficient between DL segmentations and manual labels in the original CT coordinate system, (ii) 2D Dice coefficient between DL segmentations and manual labels in three resampled SAX views (basal, middle, and apex), and (iii) Pearson correlation coefficients (ρ) between DL‐derived versus manually measured functional metrics (GLS, CS, and average WT). The 3D Dice coefficient was evaluated in the SD internal test set while the 2D metrics were evaluated in the 3D External Test Set. Both DL and manual analysis were only performed on SD images for this DL validation.

**FIGURE 2:**
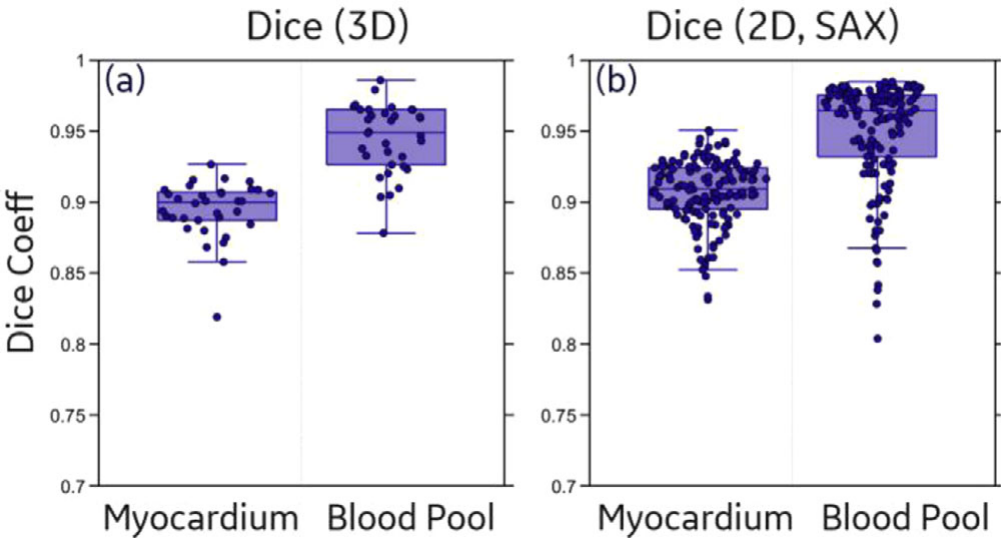
Myocardium and blood pool segmentation performance of Standard DL on SD data for DL validation. (a) 3D Dice coefficients calculated on the SD internal test set. (b) 2D Dice coefficients calculated on the SD External Test Set (at each SAX view). DL, deep learning; SAX, short axis views; SD, standard dose.

Figure 2 shows the 3D and 2D Dice coefficients between DL segmentation and manual labels. The DL network (i.e., Standard‐DL) achieves high Dice coefficients across the SD internal (3D Dice: 0.894 ± 0.020 for myocardium, 0.944 ± 0.024 for blood pool) and the SD External Test Sets (2D Dice: 0.907 ± 0.022 for myocardium, 0.949 ± 0.039 for blood pool). Higher Dice coefficients were observed for blood pool segmentation, which is likely due to the uniformly higher contrast edge over the entire volume of the LV blood pool; the posterior and septal epicardial borders of the myocardium often have low contrast.

**FIGURE 3:**
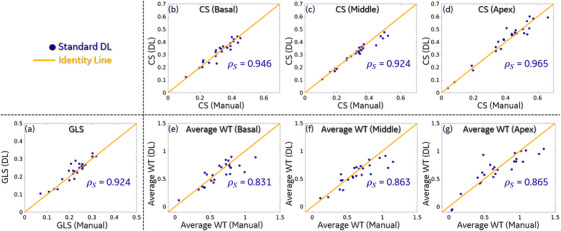
FIGURE 3 Comparison of Standard DL‐derived and manually measured functional analysis (GLS, CS, and average WT). The Pearson correlation coefficient between the Standard DL results and manual analysis is denoted as *ρ*_S. DL, deep learning; CS, circumferential strain; GLS, global longitudinal strain; WT, wall thickening.

Figure 3 shows the agreement between DL‐derived and manually measured functional metrics in the SD External Test Set. High correlation (ρ ≥ 0.92) was observed for GLS and CS measurements, indicating a good match between DL and human observers. The correlation was lower for average WT measurement (ρ ∼0.83–0.87), which is acceptable and expected considering the higher variability in WT measurement even for human observers.^17^, ^18^ We note that only Standard DL results were reported here for the sake of brevity, as Standard DL and Noise‐Robust DL have very similar performance (in terms of matching human observers) when being tested on SD data.

From the results in Figure 3 we established that properly trained DL networks can analyze functional cardiac CT data with similar results as a human observer. We then used DL as an objective observer in lower dose external test data. In particular, we are interested in determining at what dose level, the DL performance degrades to a point so that data at that dose level is not usable.

### 3.2 DL robustness for low dose images

We evaluated the performance of DL‐based functional parameter estimation at reduced dose levels with: (i) 3D Dice coefficient between DL segmentations from low dose levels and the standard dose level images, and (ii) difference between DL‐derived functional metrics (EF, GLS, CS, average WT) from low dose levels and the standard dose level. From these measurements we were able to define a threshold mA value to obtain acceptable cardiac function estimates.

**FIGURE 4:**
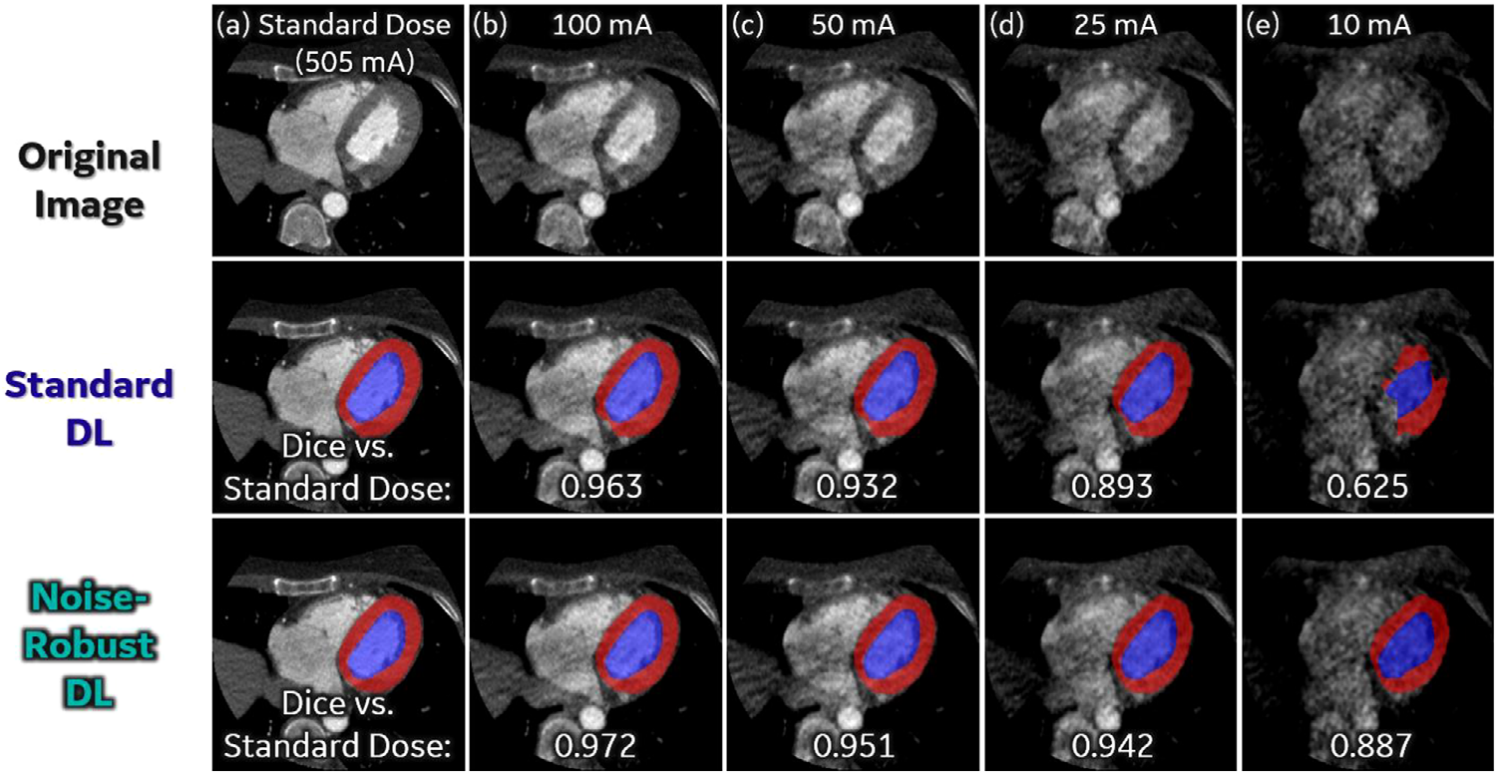
Representative DL segmentation results from Standard DL and Noise‐Robust DL at five dose levels. Column (a)‐(e): DL segmentation results overlaid on images of the corresponding dose level (standard dose, 100, 50, 25, and 10 mA, respectively). 3D Dice coefficient for the low dose segmentation versus segmentation at the standard dose level are listed below each subfigure. DL, deep learning.

Figure 4 visualizes DL segmentations on an example patient at five dose levels. When reducing the tube current to 100 mA (corresponds to ∼76.1% dose reduction on average) or even 50 mA, Standard DL provides almost identical segmentations (Dice ≥ 0.932) to those obtained from the standard dose data. When reducing the dose even further, Standard DL shows clear performance degradation as its training data consists purely of SD data. Further robustness to loss of image quality was obtained by including synthesized low‐dose data to train a Noise‐Robust DL network. Noise‐Robust DL demonstrated a much higher robustness to low‐dose data (especially at 25 mA and even 10 mA) as shown in Figure 4.

**FIGURE 5:**
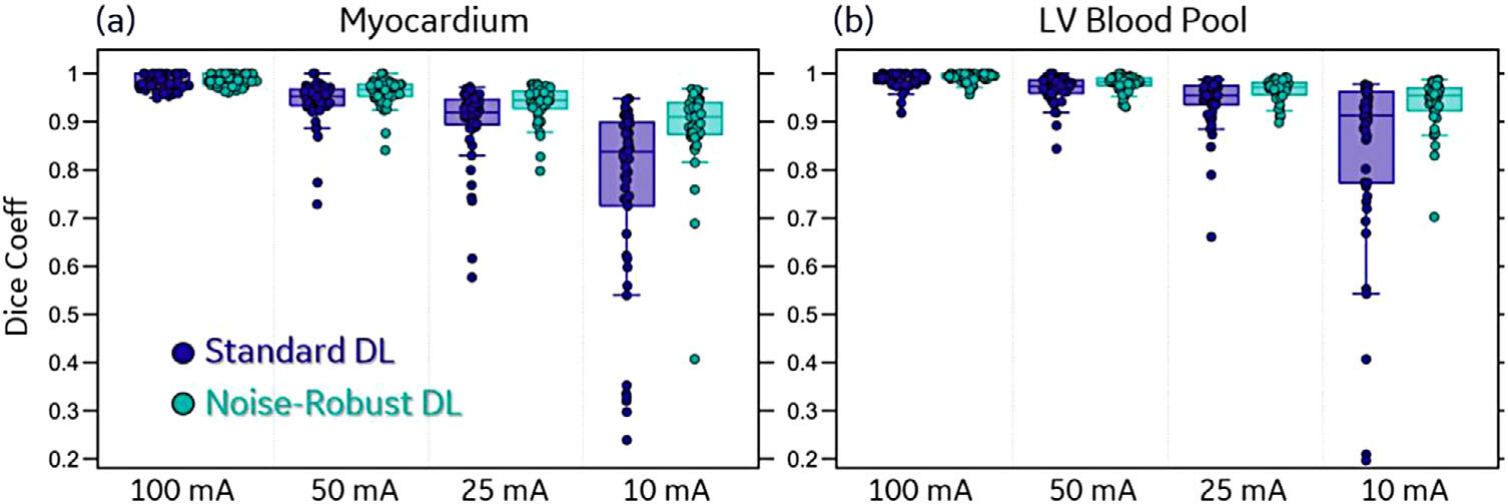
Comparison of the segmentation performance of the two networks at different dose levels. Each box plot shows the Dice coefficient between segmentation at the standard dose level and segmentation at lower dose levels (100, 50, 25, and 10 mA). (a) Dice coefficient for myocardium segmentation (red labels in Figure 4). (b) Dice coefficient for LV blood pool segmentation (blue labels in Figure 4). LV, left ventricle.

In order to measure the dose threshold of acceptable performance we compared the 3D Dice coefficient between DL segmentations on low‐dose data (100, 50, 25, and 10 mA) and DL segmentation on the same images reconstructed with standard dose data (see Figure 5). For myocardium segmentation, Standard DL achieves Dice coefficients of 0.980 ± 0.016 at 100 mA, and 0.941 ± 0.048 at 50 mA. In comparison, Noise‐Robust DL achieves Dice coefficients of 0.986 ± 0.012 at 100 mA, and 0.961 ± 0.028 at 50 mA. These high average Dice scores, and lack of any images with “failed” segmentations (i.e., boxplot outliers in Figure 5) indicate that DL segmentation exhibits minimal changes when reducing the dose from standard (∼500 mA) to 100 mA even with Standard DL. The robustness to decreased image quality was further boosted by using the Noise‐Robust DL, which exhibited minimal segmentation changes when the dose is reduced by a factor of 10 (50 mA), and performance was acceptable for most cases even with a factor of 20 reduction (25 mA). Additionally, one can see from Figure 5b that LV blood pool segmentation is slightly less sensitive to dose reduction compared to myocardium segmentation.

**FIGURE 6:**
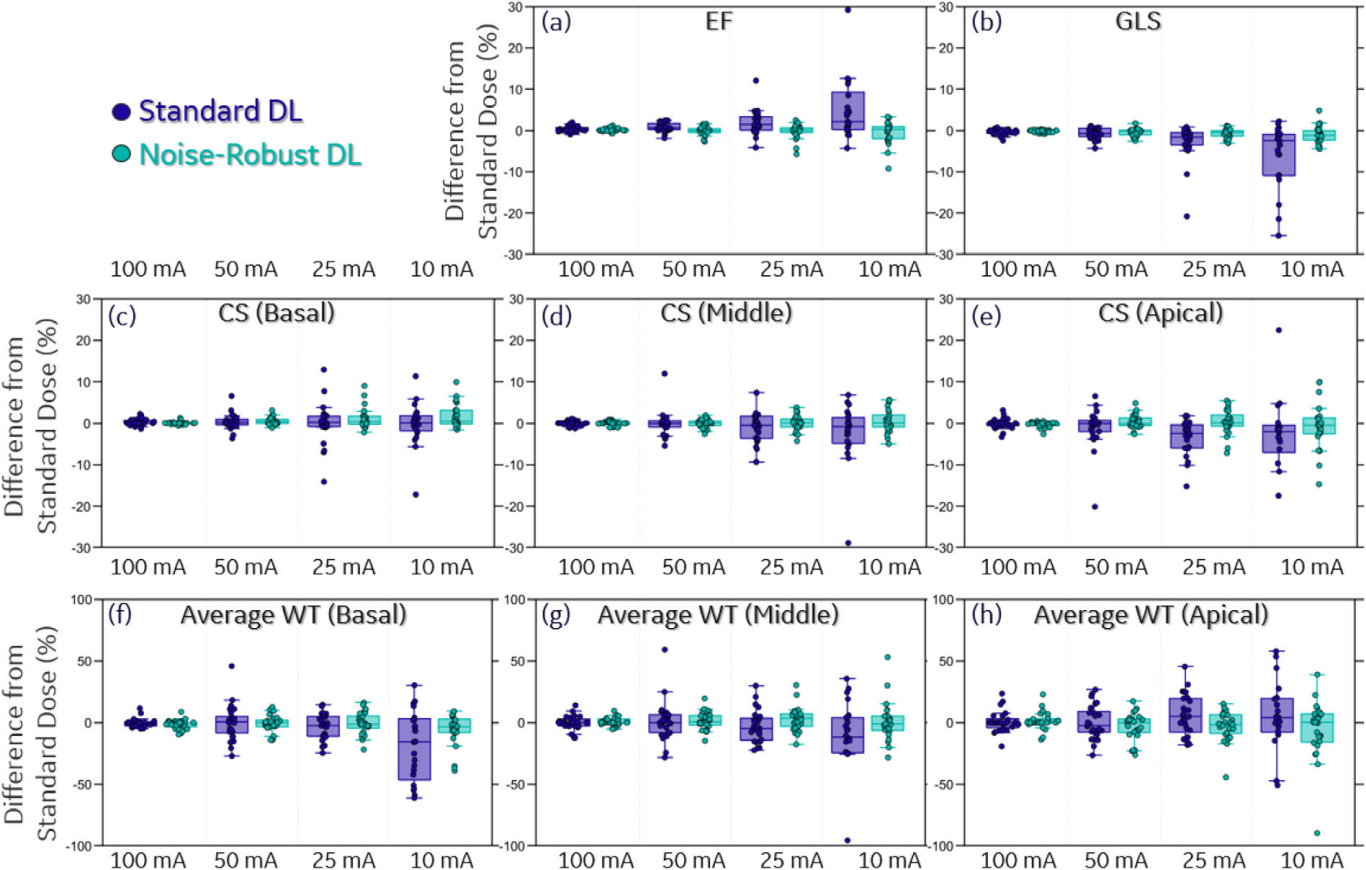
Comparison of functional analysis performance (EF, GLS, CS, and average WT) of the two networks at different dose levels. Each box plot shows the difference between the corresponding metrics obtained at lower dose levels versus that obtained at the standard dose level. Units of these metrics are all %. Therefore, the unit of the difference is also %. Overall, dose reduction to 100 mA (∼60%–80% dose reduction from the standard dose) resulted in minimal changes in functional analysis (especially EF, GLS, and CS) of left ventricular myocardium. Even lower dose level is possible if the network is also trained with lower dose data (i.e., Noise‐Robust DL). CS, circumferential strain; DL, deep learning; EF, ejection fraction; GLS, global longitudinal strain; WT, wall thickening.

Figure 6 shows the difference between functional metrics derived at low‐dose levels and the corresponding functional metrics derived at the standard dose level, which were measured for both the standard DL and the Noise‐Robust DL. These results illustrate how the DL model's functional parameter estimates change across dose levels. Function parameter estimates became more and more unstable with the decreased dose level, which is to be expected considering the image quality degradation at lower dose levels. Since these metrics (EF, GLS, CS, and WT) all have units of percent, their differences also have units of percent. At 100 mA, the absolute difference was 0.44 ± 0.54% for EF, 0.52 ± 0.85% for GLS, 0.78 ± 0.99% for CS Apical (highest among Basal, Middle, and Apical), and 5.59 ± 6.67% for Average WT Apical (highest among Basal, Middle, and Apical). The absolute difference was increased to 1.01 ± 0.82%, 1.22 ± 0.96%, 2.62 ± 4.19%, 11.12 ± 6.97% respectively at 50 mA. The robustness to decreased image quality (reduced dose level) can be further boosted by utilizing Noise‐Robust DL, which reduces the absolute difference at 50 mA to 0.72 ± 0.74%, 0.69 ± 0.70%, 1.23 ± 1.17%, and 7.82 ± 5.67% respectively for EF, GLS, CS Apical, and Average WT Apical.

These results indicate that when reducing the dose to 100 mA, Standard DL only results in minimal changes on average in functional parameters estimation for EF, GLS, and CS (with 90% percentile absolute difference ≤ 2.50%). Noise‐Robust DL allows us to further reduce the dose to 50 mA on average without introducing large changes in function parameters estimation (with 90% percentile absolute difference ≤ 2.77%). At even lower mA, performance variation of these function parameters is no longer negligible especially for larger patients (e.g., BMI > 35), this is especially the case for CS and Average WT. Estimation of WT is observed to be more sensitive to decreased image quality, which again is expected considering its higher variability even when measured by human observers.^17^, ^18^

## 4 DISCUSSION

The primary result of this work is the understanding that cardiac function analysis is possible from ultra‐low dose CT. We purposefully tested extremely low dose images to observe the dose reduction threshold where functional analysis breaks down. Our observation is that myocardial and LV segmentation failure is the key event in the functional analysis failure. We ensured that an objective observer was used to measure the loss of functional analysis quality by utilizing DL‐based segmentation techniques that were validated against expert human analysis for our patient cohort. While it was not our purpose to create a “generalized” DL network for all CT segmentation tasks, our two DL networks were excellent devices to measure the loss of quality of functional analysis as dose was reduced.

From our measurements shown in Figures 4 through 6, it is clear that radiation dose can be reduced by a factor of ∼5 (tube current reduced to 100 mA) while introducing minimal changes to the cardiac functional metrics EF, GLS, CS and WT in any of the studies investigated. The variation in clinical functional parameters found for all analyses from images using x‐ray tube current above 50 mA was found to be much smaller than the natural variability in the normal population.^19^, ^20^ As observed in other studies, the reproducibility of WT is lower than the other strain metrics.^17^, ^18^ The tube current used for this reduction (100 mA) corresponds to only ∼0.2 mSv radiation dose at 100 kV (for patient of 30 cm effective diameter, 16 cm axial scan coverage, and 0.4 s scan time to cover both ES and ED phases). The robustness of the function analysis to low x‐ray flux can be further boosted by using the emulated low‐dose data in the training set to create a “Noise Robust DL” which provides reliable functional metrics at even lower tube current (i.e., 50 mA). In many cases, especially in smaller patients, the dose can be reduced by a factor of even 20 (25 mA) while maintaining high quality segmentations and global functional estimates.

These results support the conclusion that low mA projections should be obtained in addition to the high mA projections used to construct the high‐quality coronary angiograms; these additional projections come at a very low cost in terms of dose, and high yield in terms of additional information. The exact low‐dose threshold is patent and task dependent, with patients who require high‐precision functional analysis needing high‐dose level to provide better quality images.

A fundamental assumption in this work is that segmentation is required before LV function analysis can be performed. We used the frequency of “failed” segmentations shown as outliers on the Dice score in Figure 4 and outliers in the functional parameter estimates in Figure 5 to determine if the analysis was of sufficient quality at a specific dose reduction factor. It is conceivable that one could obtain different results from DL that was trained to directly estimate strain estimates; however, the drive to further reduce x‐ray flux below 50 mA for one heartbeat is not consequential for dose reduction. However, if multiple heartbeats are needed, for example to measure myocardial perfusion, it is conceivable that tube currents below 50 mA would be useful.

Our functional estimates in dose reduced images in this work were based on emulated images for which the ED and ES image volumes were both obtained with very low mA; in reality, when a high‐quality coronary angiogram is obtained, the high‐quality diastasis or end‐systolic CCTA image can be used to assist in the functional analysis. This high‐quality CCTA image could be one image of a pair used as input to a DL network for either segmentation or direct estimates of functional parameters.

While an accurate noise injection method was already used for emulating low‐dose data, future work may include evaluation on real paired high‐dose and low‐dose data if possible. Additional future work includes training DL with more versatile training data, including cardiac patients of different body types and pathologies. We also plan to incorporate advanced data augmentation techniques to improve the generalizability of our DL observer. Despite these limitations, we believe that this work presents a proof‐of‐concept study demonstrating that radiation dose can be significantly reduced while still allowing accurate functional parameter estimates.

In conclusion, utilizing DL‐based segmentation techniques as an objective observer we demonstrate that radiation dose can be reduced by a factor of 5 (tube current reduced to ∼100 mA) while introducing minimal changes to global cardiac functional metrics (especially EF, GLS, and CS). The robustness to reduced image quality can be further boosted by using emulated low‐dose data in the DL training set, which generates a noise robust DL approach that provides reliable functional metrics at a dose reduction factor of 10.

## AUTHOR CONTRIBUTIONS

Pengwei Wu, Jed Douglas Pack, Kyle Kim, and Elliot McVeigh carried out the experiment. Jed Douglas Pack, Kyle Kim, and Elliot McVeigh prepared the patient data and label. Pengwei Wu wrote the manuscript with support from L. Severance, Jed Douglas Pack, and Elliot McVeigh. Jed Douglas Pack and Elliot McVeigh supervised the project and conceived the original idea.

## CONFLICT OF INTEREST STATEMENT

P.W.: National Institutes of Health grants; employed by GE Healthcare (job title: Research Engineer—Radiation Physics). K.K.: National Institutes of Health grants. L.L.: National Institutes of Health grants. E.M.: National Institutes of Health grants; founder shares in Clearpoint Neuro Inc., research funding from GE Healthcare, Abbott Medical and Pacesetter Inc., J.P.: National Institutes of Health grants; employed by GE Healthcare (job title: Senior Engineer—Signal Processing). In additional, P.W. and J.P. do not have fiduciary responsibility to GE Healthcare.
